# Supplementary figures and images for: Genetic Mapping of MAPK-Mediated Complex Traits Across S. cerevisiae
Source: PLoS Genet. 2015 Jan 8;11(1):e1004913. doi: 10.1371/journal.pgen.1004913 (PMC4287466; doi:10.1371/journal.pgen.1004913)

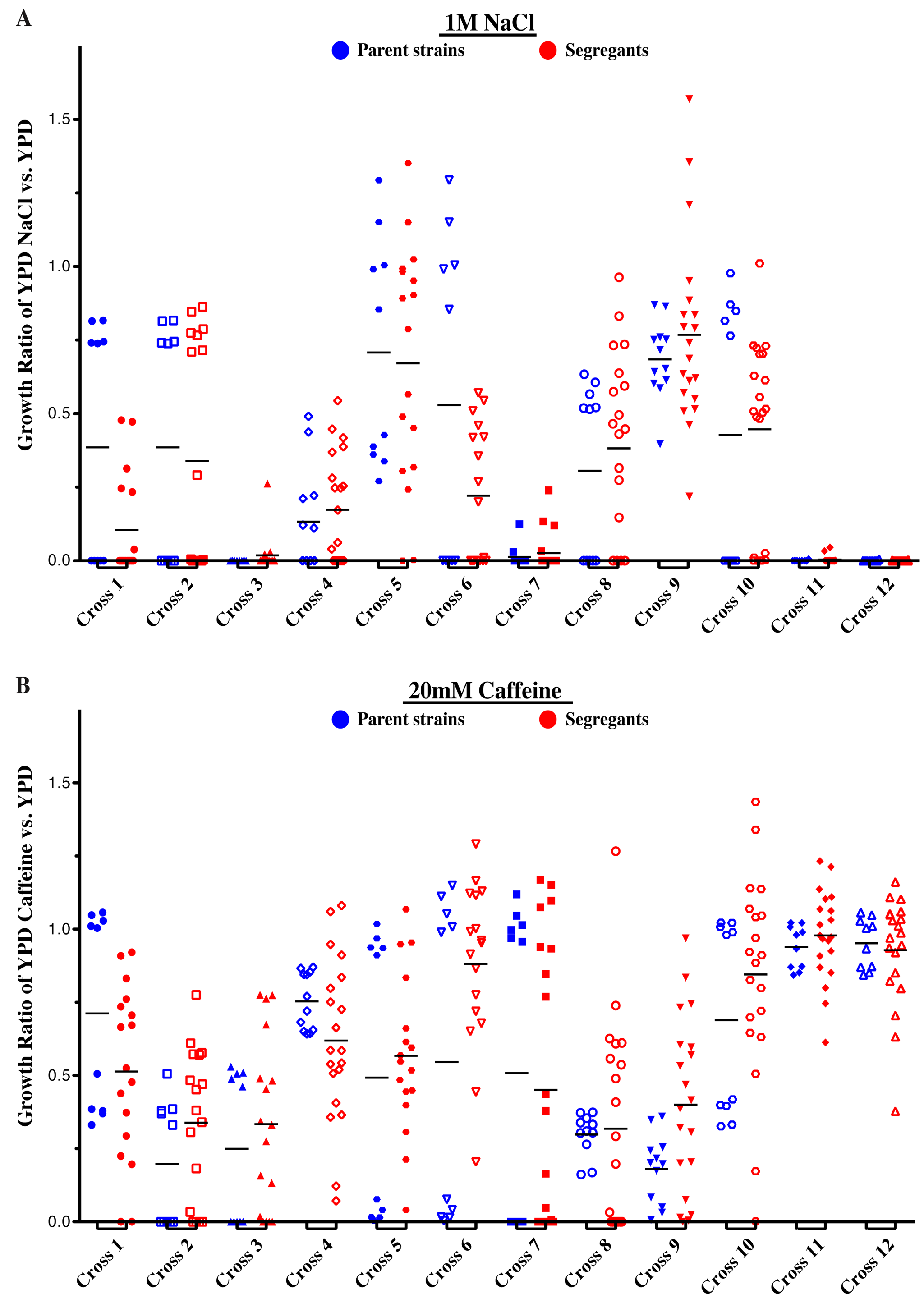

Supplement: S1 Fig — Phenotyping of individual Round Robin segregants. Between 17 and 20 recombinant haploids were isolated for each of the twelve round robin crosses and, in conjunction with the haploid parent strains, phenotyped as to their growth in the presence of (A) 1 M NaCl and (B) 20 mM Caffeine. Parent strains were phenotyped using five biological replicates. Segregants and parents of crosses 4, 7 and 9 were tested separately from those of the other crosses; hence the phenotypes of shared parent strains are not perfectly matched. Growth on the restrictive conditions was normalized using growth under the permissive YPD condition. (TIF) [file pgen.1004913.s001.tif]

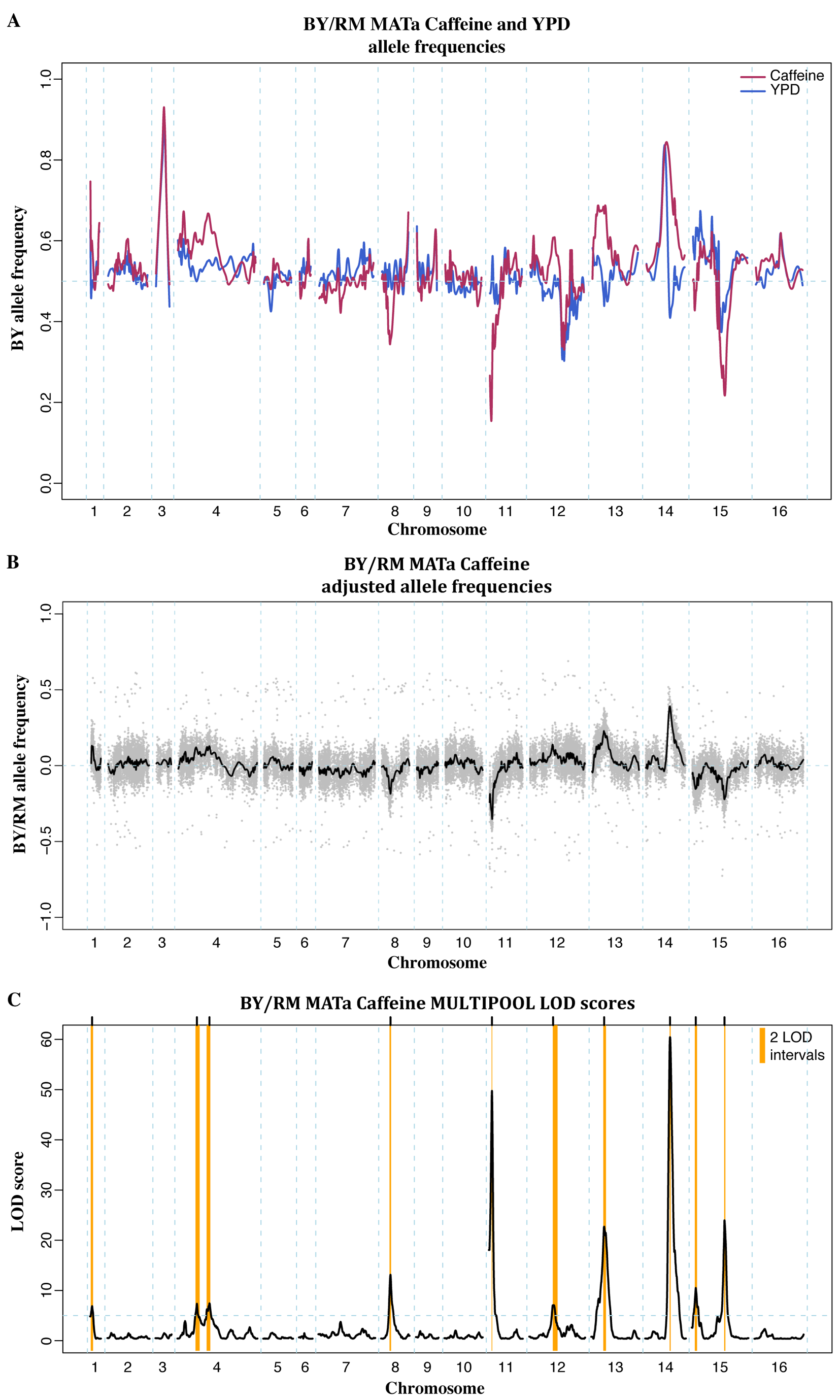

Supplement: S2 Fig — Conversion of allele frequencies to LOD scores. (A) Allele frequency plots for a BY/RM MATa caffeine selection and its corresponding YPD control experiment. Loess-smoothed BY allele frequencies across the genome are plotted. (B) To account for growth effects under the permissive YPD growth condition, allele frequencies distributions resulting from growth on YPD are subtracted from those produced by the selective condition. Grey points represent individual alleles at which allele frequencies were measured and illustrate the dense coverage of these genetic markers. (C) Allele frequencies are converted into LOD scores using the MULTIPOOL software [33]. Following the same principle as subtraction of control from selection allele frequencies, the MULTIPOOL software calculates LOD scores based on the differences between the two allele frequency distributions. QTL passing the LOD threshold of 5 (dashed horizontal blue line) are demarked on the upper axis and their 2-LOD mapping intervals indicated by orange vertical bars. (TIF) [file pgen.1004913.s002.tif]

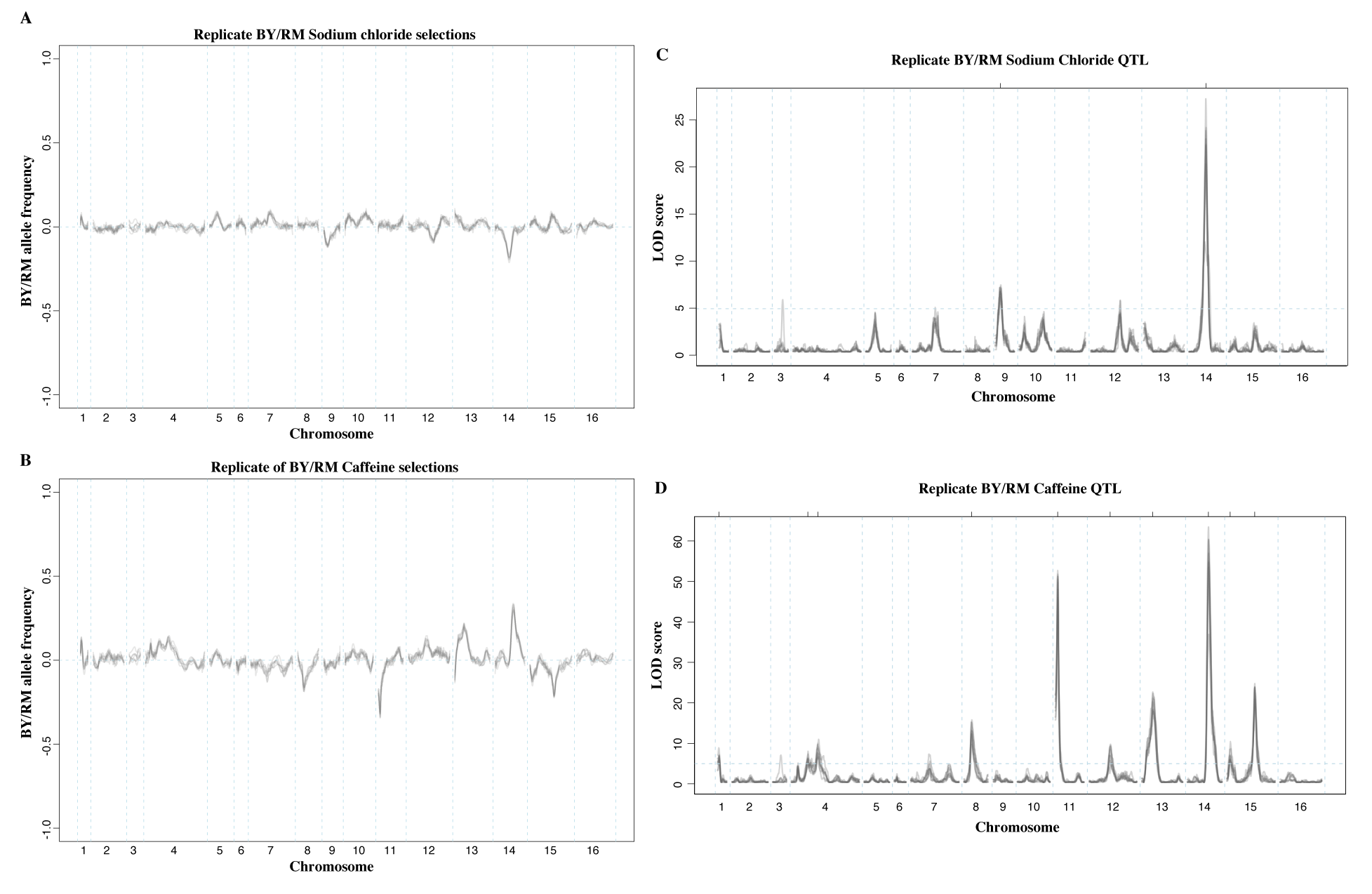

Supplement: S3 Fig — Allele frequency and QTL plots of replicate BY/RM selection experiments. Genome-wide allele frequency distributions were determined in eight replicate selection experiments each for (A) salt and (B) caffeine. The experiments were carried out in duplicate for each of two independent transformants of the BYxRM diploid with the mating type marker construct. MATa and MATα selections experiments for each provide for further replication, resulting in a set of eight replicates for each condition. Lines represent moving averages of the allele frequency spectra and are overlaid to illustrate their reproducibility. LOD score plots of replicate (C) sodium chloride and (D) caffeine selection experiments illustrate the high reproducibility of our QTL mapping approach. Each selection was carried out in quadruplicate for each mating type. The dashed horizontal line indicates the LOD threshold of 5. Tick marks on the upper axis indicate peak positions of QTL identified. (TIF) [file pgen.1004913.s003.tif]

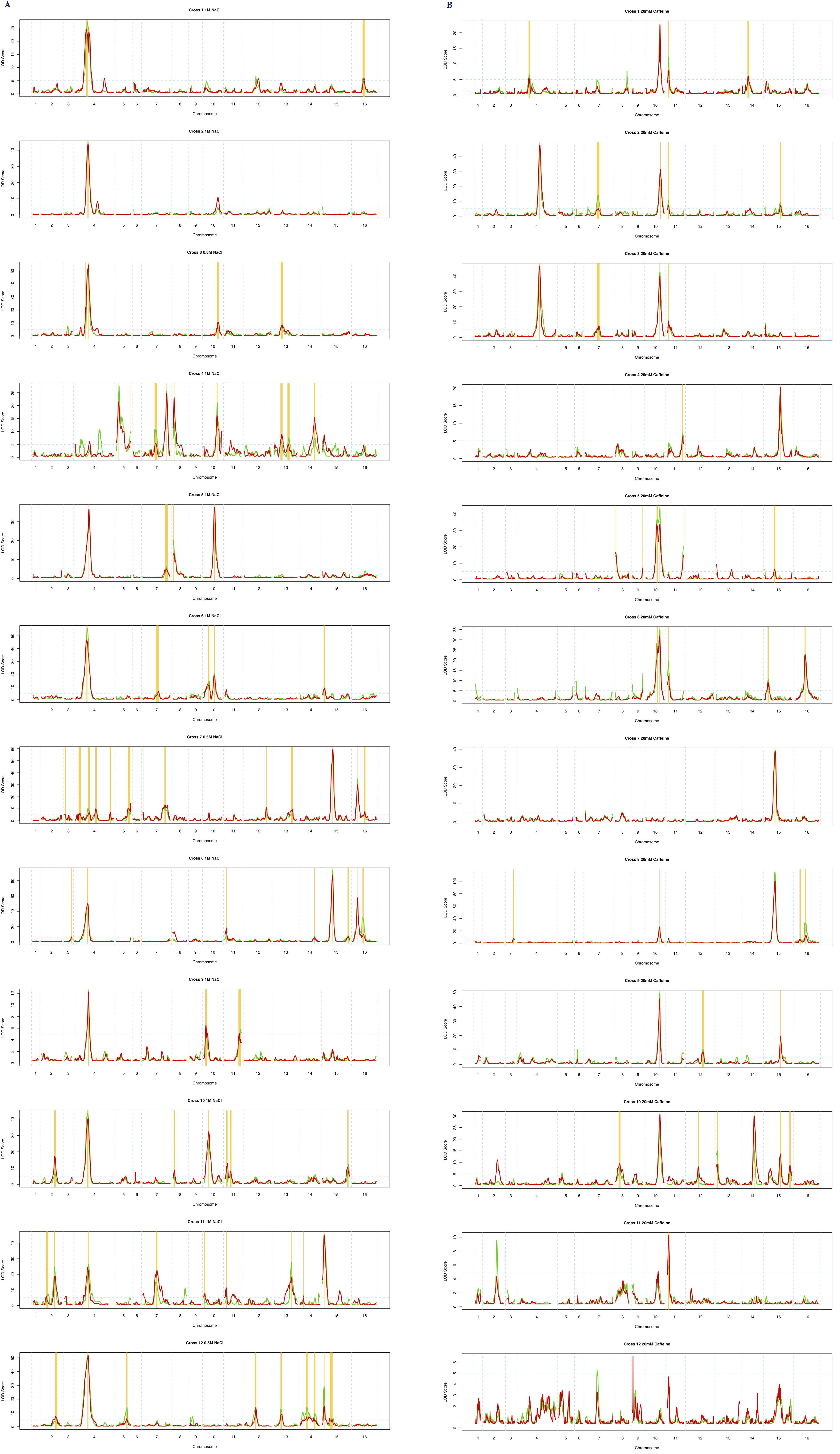

Supplement: S4 Fig — Individual LOD plots for each of the round-robin selection experiments. LOD plots of the MATa (Green) and MATα (Red) selection experiments for each cross and condition, (A) salt and (B) caffeine, are jointly plotted. The dashed horizontal line indicates the LOD threshold of 5. The 2-LOD confidence intervals of the QTL identified are indicated using vertical orange bars. For Cross 4 under the 1 M sodium chloride selection was very restrictive resulting in a low number of individual segregants in the genotyping pool and hence less reproducible LOD scores. Yet, the QTL confirmed by the replicate selection experiments were also detected using a less restrictive selection condition of 0.5 M sodium chloride and largely (7 of 9 QTL) shared with other crosses. (TIF) [file pgen.1004913.s004.tif]
